# Sound symbolism is not “marginal” in Chinese: Evidence from diachronic rhyme books

**DOI:** 10.1371/journal.pone.0322044

**Published:** 2025-05-14

**Authors:** Yingying Meng, Yuwei Wan, Chunyu Kit

**Affiliations:** Department of Linguistics and Translation, City University of Hong Kong, Kowloon, Hong Kong SAR, China; University of Kurdistan Hewler, IRAQ

## Abstract

Contrary to the widespread notion that linguistic signs are arbitrary, researchers have consistently demonstrated the existence of sound symbolism in language, providing evidence for non-arbitrariness in sound-meaning associations. However, much evidence of this kind is based on a limited subset of vocabulary and falls short of systematically demonstrating the pervasive nature of sound symbolism and, especially, its central, rather than marginal, role in language. Furthermore, a historical perspective is lacking to determine whether sound symbolism is merely a feature of archaic languages or has remained a significant element throughout the evolution of languages. This research pioneers a diachronic analysis of sound symbolism in Chinese using historical rhyme books to trace its presence on the vocabulary scale. Employing natural language processing techniques along with statistical methods, it investigates whether phonologically related Chinese characters, as documented in rhyme books, also demonstrate semantic congruence, which would suggest that the phonological aspects of characters are inherently meaningful and hence indicate a systematic, rather than random or purely arbitrary relationship between sounds and meanings. Statistically significant results from our analysis of all four analyzed rhyme books confirm the robustness of sound symbolism over a large span of the Chinese language continuum, and a granular analysis of a representative one of them further reveals that sound symbolism is manifest across various levels of phonological organization, including initials, finals, etc. This study initiates an innovative combination of traditional materials with novel techniques to enrich and expand existing knowledge about sound symbolism, providing both methodological advancement and empirical insights.

## Introduction

Sound symbolism, also known as phonetic symbolism or phonosymbolism, is often described as the non-arbitrary association between speech sounds and the meanings they convey. This association could be either natural mimesis (iconicity), where sounds inherently resemble their referents, or learned associations including language habits that link specific phonetic elements with particular semantic traits [1]. Substantial research has been conducted on sound symbolism, confirming its pervasive presence across languages [2–4] and across various semantic dimensions such as size, shape, lightness, temperature, and emotional resonance. Specifically, Sapir’s [5] experiments demonstrated magnitude symbolism associated with vowels and consonants. Köhler [6,7] discovered the bouba/kiki effect, illustrating the intuitive connection between sounds and shapes. Newman [8] explored the symbolism between sound and visual attributes such as lightness and darkness and linked them to physical articulation properties. Whorf [9] added dimensions like temperature, correlating specific vowels with sensory attributes like warmth and coldness. Whissel’s [10–12] studies on the emotive use of phonemes in texts revealed systematic variations in phoneme usage corresponding to emotional content. Kraxenberger et al. [13] validated the influence of prosodic features like pitch and rate on emotional interpretation, indicating that even without semantic understanding, the emotional undertones of sound are perceptible across languages.

Building on extensive evidence confirming the existence of sound symbolism, recent studies have moved to explore its pivotal role in language evolution, acquisition, and processing. Ćwiek et al. [14] demonstrated, via an online study involving 917 participants from 25 languages and 10 writing systems, that the bouba/kiki effect is robust and extends across cultures, independent of orthographic influences, suggesting that crossmodal sound-symbolic mappings may have actively contributed to shaping the evolution of spoken language vocabularies. Imai and Kita [15] proposed the sound symbolism bootstrapping hypothesis, arguing that non‐arbitrary sound-meaning correspondences enable pre‐verbal infants to gain referential insight and establish lexical representations, thereby facilitating word learning and potentially influencing language evolution through iconic bootstrapping. Bergen [16] explored the psychological reality of English phonesthemes by showing that recurring sound‐meaning pairings, such as the “gl-” onset in words related to light and vision, produce facilitatory priming effects similar to those observed for morphologically related words. These findings challenge strictly compositional models of morphology and instead support usage‐based accounts, suggesting that the frequency and statistical prevalence of recurring form‐meaning pairings play a critical role in lexical organization and language processing. Farmer, Christiansen, and Monaghan [17] demonstrated that phonological typicality—the degree to which a word’s sound conforms to the phonological patterns typical of its lexical category—facilitates on-line sentence comprehension, as words with more typical sound forms are processed more efficiently. Their corpus analyses and priming experiments indicate that non-arbitrary, statistically frequent sound-meaning associations contribute to real-time language processing.

Many of these studies have employed an experimental approach in which symbolic meanings were evaluated by human participants, often through tasks where participants deduced the meanings of words or pseudo-words based on pronunciation. Human evaluation provides valuable insights into sound symbolism by capturing intuitive responses to phonetic cues. However, it also introduces variability due to individual perceptions, cultural backgrounds, and personal experiences, which may impact the reliability of the findings. The use of pseudo-words in some studies helps to control for pre-existing lexical meanings, but participants may still associate pseudo-words with similar-sounding real words, which inevitably influences their judgments in an unexpected way. For instance, pseudo-words like “juy” and “flike” might evoke associations with words like “joy” and “like,” which carry strong emotional connotations, resulting in high emotion intensity ratings [18]. With the development of corpus tools and natural language processing (NLP) techniques, word vectors are being employed as a new way of gauging meanings and further computing semantic similarities, and machine learning helps dig into the importance of specific phonemes in meaning prediction. Otis and Sagi [19] used Infomap for word vector representation, singular value decomposition for dimension reduction of the vectors, and cosine similarity to measure their semantic relationship. Abramova et al. [20] used the Semantic Vectors package to build a distributional semantic model based on the British National Corpus and obtained results that augmented Otis and Sagi’s previous study; they further used WordNet to automatically identify the semantic content of some of the phonesthemes. Abramova and Fernández [21], building on the above two studies, used 400-dimensional word embeddings as representations of word meanings and adopted a stricter phonestheme validation procedure, including a more challenging baseline, and attempted to derive the meanings of the phonesthemes using fully unsupervised methods. Sabbatino et al. [18] trained emotion intensity prediction models with character embeddings and phoneme embeddings and determined the potential of word pronunciation in predicting emotion intensity.

While sound symbolism as a concept has been extensively documented in linguistics, existing research still faces critical constraints that limit its explanatory scope. These include the phonological complexity of alphabetic languages and the lack of reliable records of ancient speech sounds. Many alphabetic languages, such as English, feature complex phonological structures with words often consisting of multiple syllables. This complexity makes clear-cut phonological categorization difficult, as categorizing based on individual syllables or phonemes can overlook the influence of additional phonological elements in the same semantic unit. Consequently, statistical methods are necessary to disentangle such influence and isolate significant sound-meaning associations [22]. Besides, as the auditory attributes of many ancient languages are unrecorded, existing sound symbolism studies are concentrated on modern languages and the exploration of symbolic meanings is relegated to interpretations derived mostly from contemporary respondents. However, there is a belief that although many names of objects and events originally arose from a resemblance between visual or tactile qualities and certain sounds or acoustical wholes, the connection of this kind has been largely lost in contemporary languages [6]. A diachronic study is hence required to determine whether the sound symbolism characteristic of language is disappearing or remains a prominent feature all the time. Our work builds on the rich theoretical foundation of sound symbolism but shifts focus to a historically understudied dimension: how sound symbolism operates and evolves in a logographic system like Chinese, leveraging rhyme books as structured diachronic corpora.

Chinese provides unique opportunities for sound symbolism studies due to two key factors. On the one hand, Chinese exhibits minimal morphological complexity, with a substantial proportion of its characters being monosyllabic morphemes. That is, many Chinese characters embody a direct amalgamation of sound and meaning, enabling intuitive investigation of sound-meaning correlations. This is further facilitated by the high prevalence of homophones in Chinese, which allows for a straightforward exploration of these relationships. On the other hand, Chinese *yinyun xue* 音韻學 (phonology), which primarily focuses on the pronunciation of Chinese characters and their changes across different historical periods, has evolved over a thousand years and preserved a wealth of valuable historical phonological information, offering an invaluable set of materials for studying etymology and language development [23]. As carriers of Chinese phonology, rhyme books are sound-based, dictionary-like compilations that systematically categorize the pronunciation patterns of characters. Through these compilations, the phonological evolution of the Chinese language can be analyzed and traced in detail, allowing a diachronic investigation of sound-meaning relationships. Compared with many prior studies that were conducted on a small, well-designed set of words or pseudo-words, our research employs rhyme books that cover most Chinese characters or at least the frequently used ones, enabling an objective examination of sound symbolism truly on the general vocabulary scale. Additionally, certain rhyme books provide detailed records of character pronunciations, including initials, finals, tones, and rhyme categories, allowing for an in-depth investigation into which sound features carry symbolic significance. Utilizing these materials, sound symbolism can be explored in greater breadth and depth through both longitudinal and fine-grained analyses.

Our study introduces a novel framework that synergizes the phonology in historical rhyme books with computational methods to trace sound symbolism diachronically—an understudied dimension in prior research. Central to this approach is the integration of distributional semantic embeddings, which objectively encode character meanings based on contextual usage patterns in large corpora. These embeddings are used in place of subjective human evaluations, ensuring scalable, bias-resistant analysis of semantic relationships across tens of thousands of Chinese characters. Building on the pairing of phonological groupings in rhyme books with character embeddings, three complementary computational methods are employed in this study: (1) cosine similarity to quantify semantic proximity within a phonological group, (2) statistical tests to validate significance against baselines, and (3) visualization tools to map clusters of sound-meaning associations. Combined, these methods allow for broadly tracking sound-meaning relationships across rhyme books while drilling down into specific phonological units or even particular homophone groups. To address the limitation of reliance on only synchronic or anecdotal evidence, as found in many prior studies, our approach leverages the longitudinal structure of rhyme books to systematically decode sound symbolism across different time periods and at multiple granularities (e.g., initials, finals, and rhyme categories). The result is a replicable, data-driven paradigm that not only bridges computational linguistics and historical phonology but more importantly, demonstrates how historical resources can be leveraged to interrogate modern theories of language cognition.

Drawing from these unique linguistic and philological resources and novel techniques, this study addresses three key questions. First, how pervasively does sound symbolism manifest in Chinese characters, and how has it evolved diachronically across recorded phonological history? Second, at what phonological granularity do sound-meaning associations manifest, and in which phonological units (e.g., initials, finals, rhyme categories) can these symbolic relationships be observed? Third, how do phonetic features influence the semantic interpretation of Chinese characters?

The remainder of this article will first move on to detailing the data that are utilized in this research, including the four rhyme books and the corresponding sets of character embeddings. Then, the methods to analyze semantic similarities among characters within the same phonological group are outlined, including calculating cosine similarities between character embeddings, computing the average semantic similarity of a phonological group, performing statistical analysis of group similarities, and visualizing the data using plotting methods. After that, we present our findings, offering a diachronic analysis of the general trends and patterns of sound-meaning associations, a focused study of diverse phonological units, and an exemplary illustration of how phonologically related characters are distributed in a semantic vector space. Finally, we conclude with a summary of our key findings, the significance and limitations of this research, and possible future research directions.

## Data

The data for this study was drawn from two primary sources: Chinese rhyme books, which provide phonological information of characters, and character embeddings, which represent the semantic properties of characters in a multidimensional vector space.

### Rhyme books

Rhyme books (*yun shu* 韻書), also called sound books (*yin shu* 音書) are books that record Chinese character pronunciations. Compiled mainly for the purpose of facilitating the writing of rhymed compositions (such as poetry, lyrics, etc.), they are continually updated to keep pace with the evolution of the pronunciations of Chinese characters. Despite the complexity of speech sounds due to dialectal variations and the evolving nature of language, rhyme books from specific historical periods still largely capture the general phonological features of the time. Organized into character groups based on shared phonological features, they cover most characters or at least the frequently used ones in the language.

#### Guangyun.

The earliest extant rhyme book in China is *Qieyun* 切韻, a rhyme book using *fanqie* 反切, a sinographic spelling method, to indicate character pronunciation [24]. It clearly presents the system of initials, finals, and tones of the Chinese language from the Sui dynasty (581–618 AD) to the early Tang dynasty (618–approximately 907 AD) and essentially represents the phonological system of Middle Chinese [25]. Since it was lost for long and only its fragments were found in the 1930s [23], its expanded edition *Guangyun* 廣韻 (extended rhymes, hereinafter *GY*), which basically keeps its phonological system and incorporates more characters and annotations, has become the most representative rhyme book of Middle Chinese and a preferred resource for studying its phonological system [25].

*GY* is organized in a hierarchical structure; that is, characters having the same pronunciation (initial, final, and tone) form a *xiaoyun* 小韻 (small rhyme) group, small rhymes sharing the same nucleus, coda, and tone fall under the same *yunmu* 韻目 (rhyme heading), and rhyme headings with the same coda and similar nuclei form a *yunshe* 韻攝 (inclusive rhyme) group, each of them being represented by a Chinese character.

We obtained the digital version of *GY* from the website Yundianwang 韻典網 (https://ytenx.org/, hereinafter YDW) and curated it with the printed version *Song Ben Guangyun* 宋本廣韻 (Song edition of the extended rhymes) [26]. The latter contains 206 rhyme headings, but YDW supplemented four more, resulting in 210 altogether. YDW also provided the initial, final, and tone of each small rhyme. Since characters in the same small rhyme group have identical pronunciations, we can divide all the characters in *GY* into different initial, final, and tonal groups. There are different opinions on how many initials there are in *GY*, and YDW adopted the 37-initial system of Shao Rongfen 邵榮芬 but added a separate *ɣ* 云 initial, leading to 38 initials. Based on the nuclei, YDW divided some finals into finer groups, but to avoid complicating the picture, we only adopted the broader division, namely 96 finals altogether for this research. That is, the characters in *GY* were assigned to 3,874 small rhymes, 210 rhyme headings, 16 inclusive rhymes, 38 initials, 96 finals, and 4 tones, allowing the semantic similarities of phonological groups under these division schemes to be examined separately.

#### Pingshui yun.

Over time, the *GY* system diverged increasingly from contemporary speech, creating challenges for scholars and especially poets who relied on it in literary composition, which necessitated the emergence of *Pingshui Yun* 平水韻 (rhyming system of Pingshui, hereinafter *PSY*) in response. *PSY* is not the title of a specific rhyme book, but a reference to the 106-rhyme system, which significantly reduced the number of rhymes from over 200 in the *GY* system. During the Yuan dynasty, the composition of regulated verse required adherence to *PSY*, a tradition that has persisted to the present [25].

We included *PSY* in our diachronic study for several reasons. First, it continued to be relevant across multiple dynasties and acts as a cross-generational reflection of phonological conditions. Second, due to its adoption in imperial examinations and its usage in literary compositions, it exerted a significant influence on literary Chinese and helped standardize poetic forms across diverse linguistic regions and periods. This widespread usage makes it an essential subject of study for understanding the trends and shifts in Chinese phonology. Third, it simplified and merged the earlier more complex systems into a manageable framework and summarized the overarching patterns and general rules, providing insights into the broad phonological landscape of the Chinese language.

The 106 rhymes in *PSY* are arranged in four sections by tone. The digital version of *PSY* was obtained from the website Souyun 搜韻 (https://sou-yun.cn/QR.aspx).

#### Zhongyuan yinyun.

In the Yuan dynasty (1271–1368 AD), metrical rules were followed in the composition of Yuan Qu 元曲 (Yuan drama). Since significant changes in pronunciation had occurred from Middle Chinese to early modern Chinese, *GY* could no longer accurately represent the actual phonological system of that time. Zhou Deqing 周德清 revolutionarily derived a phonological system from the actual spoken language and the rhyming rules in the Yuan Qu works and composed *Zhongyuan Yinyun* 中原音韻 (phonology of the central plains, hereinafter *ZYYY*), which became a representative rhyme book of early modern Chinese pronunciations [25].

*ZYYY* comprises 19 rhyme groups, each represented by two characters used as rhyme headings. Each group includes characters in the level, rising, and departing tones; level tones are further divided into *yin* 陰平 and *yang* 陽平. Within the same tone, characters are divided into various homophone groups, also known as small rhymes. The original book incorporates a total of 5,866 characters, and its digital version is also available on Souyun.

#### Zhonghua tongyun.

In the past century, the Chinese language has witnessed noticeable transformations in pronunciation, paralleled by the simplification of the written form of characters and the promotion of Putonghua as the standardized national language. These changes were fueled by the early 20th-century vernacular movement, which aimed to bring written Chinese closer to its spoken form in the hopes of facilitating clearer communication. Given this evolution, traditional rhyme books, rooted in historical linguistic frameworks, do not adequately reflect contemporary pronunciation patterns. In 2019, *Zhonghua Tongyun* 中華通韻 (common rhyme of Chinese poems, hereinafter *ZHTY*) was officially released as an authoritative and practical rhyme book for contemporary poetic creations. It is based on the phonological foundation of Putonghua, currently in common use in China, and covers standardized simplified Chinese characters, providing a practical resource for exploring the phonological aspects of modern Chinese.

*ZHTY* incorporates 7,730 Chinese characters under 15 rhyme groups, each of which is further divided into four subgroups based on tones. The digital version of *ZHTY* is also available on Souyun.

### Character embeddings

Word vectors, or embeddings, represent words as multi-dimensional numerical arrays, capturing the semantic properties of words based on their usage in texts to facilitate text processing by machines. They can be applied in numerous machine learning tasks, ranging from semantic analysis to emotion intensity prediction. Word embeddings can be divided into three categories depending on how they are generated, namely traditional frequency-based word embeddings, static word embeddings, and contextualized word embeddings [27]. The static ones are often used for the computation of semantic similarity between words through metrics such as cosine similarity, which measures the cosine of the angle between two vectors in space. The most widely adopted methods for training static word embeddings include the statistical model GloVe [28] and the neural network-based Word2Vec model, which uses either the Continuous Bag of Words (CBOW) or Skip-gram architecture [29,30].

As mentioned above, many Chinese characters are monosyllabic morphemes that directly combine sound and meaning. Even when a character is part of a multi-character word, character embeddings still capture its semantic contribution. Therefore, we prioritize character embeddings over word embeddings to maintain the character-level focus of this study and to examine the unique sound-meaning relationships intrinsic to individual Chinese characters. The selection of embeddings to represent character meanings is of paramount importance to our methodology. Due to the unavailability of period-specific embeddings for each of the four rhyme books, we opted for two primary sets of embeddings from the same source named Chinese Word Vector (https://github.com/Embedding/Chinese-Word-Vectors) [31]. The two embedding sets were trained using the Skip-Gram with Negative Sampling (SGNS) model [29,30] and a ngram2vec [32] toolkit. The basic settings for the SGNS model included a window size of 5, dynamic window enabled, a sub-sampling rate of 1e-5, a low-frequency word threshold of 10, five iterations, and negative sampling set to 5. The vector size was set to 300. The consistency in training methods and settings ensured that the embeddings were methodologically comparable. The first set of embeddings was trained on *Siku Quanshu* 四庫全書 (complete library in four sections, hereinafter *Siku*), the largest collection of texts in pre-modern China. Two versions of *Siku* embeddings were provided in the Chinese Word Vector dataset, and we opted for the one trained with individual Chinese characters instead of bigrams as context features. The second set of embeddings was trained on Baidu Baike (https://baike.baidu.com/, Baidu encyclopedia), which serves as a representative corpus for training modern Chinese word embeddings because of its comprehensive and contemporary content. Using this corpus, the developers [31] trained embeddings for various target and context units, based on different co-occurrence statistics, such as Word → Word, Ngram (1–2) → Ngram (1–2), and Word → Character (1). Since we needed character representations, we adopted the character vectors developed from the “Word → Character (1)” type, with characters as the context units. A key factor in selecting these two sets of embeddings is that they were trained on two extensive, comprehensive, and representative corpora, ensuring their capacity to accurately capture the semantic relationships between characters.

The *Siku* embeddings were used for the first three rhyme books *GY*, *PSY*, and *ZYYY* while the Baidu embeddings were applied to *ZHTY*. We assumed that the *Siku* embeddings, derived from classical texts from the recorded history of China up to the mid-Qing dynasty, were generally applicable to the three pre-modern rhyme books, and the Baidu embeddings, which reflect contemporary language usage, were suitable for the rhyme book *ZHTY* compiled to guide poetry creation in modern times. As posited by Wang [33], semantic shifts were typically gradual rather than abrupt. That is, semantic evolution is continuous, and consequently, the language used in earlier periods remains mostly comprehensible to speakers in later periods. Although the meanings of some characters in the texts of *Sik*u may slightly vary across time, the embeddings trained on *Siku* can still generally represent these meanings, especially regarding the semantic relationships between characters. However, significant changes in the Chinese vocabulary occurred during the two to three decades following 1919 [33], making it beneficial to adopt a new set of embeddings.

To further check whether the findings based on *Siku* embeddings were valid to ensure the robustness of our conclusions, we trained three more sets of embeddings and conducted a focused study on *GY*. The decision to train additional embeddings specifically for *GY*, rather than *PSY* and *ZYYY*, was driven by several factors. First, *PSY* covers a broad spectrum of materials that are challenging to gather and verify. Second, while *ZYYY* is primarily relevant to Yuan Qu, *GY* is extensively utilized in Tang poetry composition, which places significant emphasis on sound—an essential element of poetry. Additionally, the contemporary materials for *GY* are readily accessible online. Therefore, to optimize the effectiveness of our study, it is reasonable to concentrate on *GY* for verifications. Specifically, considering that *GY* was widely used to guide poetry creation in the Tang dynasty, we used *Quan Tang Shi* 全唐詩 (complete collection of Tang poems, hereinafter *QTS*) to train a set of character embeddings. In case the *QTS* corpus is not large enough or is limited by text genres to generate reliable meaning representations, we trained two more sets of embeddings, one using *Quan Tang Wen* 全唐文 (complete collection of Tang proses, hereinafter *QTW*), and the other using a combination of *QTS* and *QTW*. A similar SGNS model to the one mentioned above was used to train these three sets of embeddings. However, due to the relatively smaller corpus sizes, the model was slightly adjusted with the following parameters: vector_size = 100, min_count  = 1, and sample = 1e-3.

## Methods

The main idea of our methodology is to see whether characters belonging to the same phonological group also tend to have closer-than-average meanings. To achieve this goal, cosine similarity was used as the metric to gauge semantic similarities, statistical testing was conducted to determine the significance of any divergence from baselines, and suitable plotting methods were used to visualize the data and present the results in an intuitive way. The code, text data, and embedding datasets required to replicate this study are available at https://figshare.com/s/6d9fdbdf4b3f164b930d. The collection and analysis of the data adhered to the terms and conditions of respective data sources.

### Semantic similarity measurement

Cosine similarity is a widely used metric in natural language processing to quantify the similarity between two non-zero positive vectors by measuring the cosine of the angle between them [34]. Mathematically, cosine similarity between two vectors A and B is defined as:


$$Cosine\;Similarity=\;\frac{A\cdot B}{\left||A||\;||B|\right|}\mathrm{\backslash ]}$$

where A·B represents the dot product of the vectors, and ||A|| and ||B|| are the magnitudes of the vectors. This measure focuses on the direction of the vectors rather than their magnitudes, making it particularly useful for applications where the relative orientation of vectors is more important than their lengths. It can be used to measure “the similarity between a query and a document, between two documents, or between two terms” [35], and therefore is widely adopted in information retrieval and document clustering. In this study, with characters converted to vectors, we adopted cosine similarity as the metric to measure the semantic relatedness between two characters. We utilized the “cosine_similarity” function from the scikit-learn library in Python to compute these similarities.

**Fig 1:**
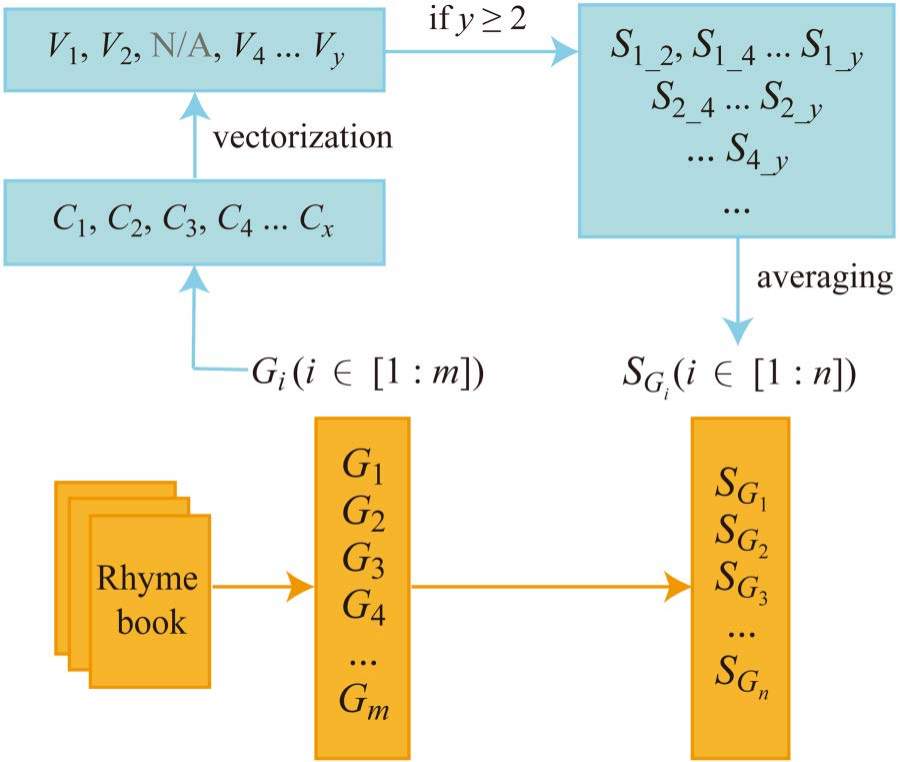
Workflow of obtaining semantic similarities of phonological groups in a rhyme book.

The process of obtaining semantic similarities of phonological groups is illustrated in Fig 1. Specifically, the characters in a rhyme book were divided into phonological groups, each group *G*_*i*_ (*i* ∈ [1: *m*]) being composed of characters *C*_1_, *C*_2_, …, *C*_*x*_, which were vectorized into *V*_1_, *V*_2_, …, *V*_*y*_. Since the training materials for the embeddings were comprehensive and large-scale, they covered most of the characters in the rhyme book. However, some characters, particularly rare ones, might still be missed, resulting in *x* ≥ *y*. This was acceptable because the missed characters were typically rare, constituting only a small portion of the total characters. At least two items were required in a phonological group to calculate the pairwise cosine similarity, so only when *y *≥ 2 was the “cosine_similarity” function applied to a group to obtain its pairwise similarity matrix. Then, to extract the unique similarity values between different items, only the upper-triangular part of the matrix for all unique pairs, excluding the diagonal, was retrieved and flattened into a 1D array, resulting in a list of cosine similarities, such as *S*_1_2_, *S*_1_*y*_, … in the figure. Averaging all these pairwise cosine similarities yielded the group semantic similarity, represented as $S_{G_{i}}$ (i ∈ [1: *n*]), where *n* ≤ *m* since some groups with *y *< 2 were excluded. The characters covered by the final groups constituted a population, and the average cosine similarity over all pairs of characters in this population was taken as the overall semantic similarity of the rhyme book.

### Visualization of semantic similarities and statistical significance testing

We compared the semantic similarity of each phonological group with the population mean to determine if it is greater than average. Additionally, to evaluate whether the high similarities occurred by chance, we created comparison groups by randomly sampling from the population, with each group containing the same number of characters as the original to eliminate possible effect of group size on the group semantic similarity. To examine how the semantic similarities of phonological groups and comparison groups were distributed, we utilized violin plots for a comprehensive overview of data distribution, including density, range, and central tendency. Comparing the median line and the population mean of each violin plot would give a straightforward answer to whether the group semantic similarity tend to be greater than expected.

Statistical testing was employed to assess the significance of the divergence, if any. We used the non-parametric one-sample Wilcoxon signed-rank test, which involved calculating the difference between each group’s semantic similarity and the population mean, and then testing if the median of the differences significantly differed from zero. In a similar task of determining phonesthemes, Abramova and Fernández [21] conducted independent-samples one-tailed Welch’s t-tests, comparing the pairwise similarity of a phonestheme to that of the corresponding baseline cluster. We did not follow this to use Welch’s t-test because, unlike the carefully selected phonestheme groups, many of our naturally formed groups in rhyme books are too small (containing fewer than 30 samples) to meet the assumptions required for Welch’s t-test, particularly normality or sufficient sample size. The non-parametric one-sample Wilcoxon signed-rank test does not require these assumptions, making it a more appropriate choice for our analysis. We used the “stats.wilcoxon” function from the “scipy.stats” Python package for this calculation.

However, the Wilcoxon signed-rank test requires at least five groups, making it unsuitable for examining the four tonal groups. Therefore, we adopted a Monte Carlo simulation approach similar to the one used in [19] to assess the semantic coherence of tonal groups. This approach is feasible because, unlike some other phonological groups with very few characters, the four tonal groups each contain a sufficient number of characters to allow for robust statistical analysis.

### Dimension reduction and visualization of character vectors

In this study, high-dimensional character embeddings were adopted to represent the semantics of characters. To show how characters sharing common phonological features might be distributed in a two-dimensional semantic space, dimension reduction was required. Principal Component Analysis (PCA) [36] and t-distributed Stochastic Neighbor Embedding (t-SNE) [37] are two widely adopted dimension reduction methods, while Uniform Manifold Approximation and Projection (UMAP) [38] is a recent manifold learning method. PCA reduces dimensionality by projecting data onto directions of maximum variance but struggles with nonlinear relationships common in language data. Non-linear methods like t-SNE and UMAP address this issue. While t-SNE preserves local structure, UMAP excels at maintaining both local and global structures [39]. Since our goal was to visually confirm whether characters from the same phonological group would cluster together in the plot, we adopted UMAP for its flexibility and excellent performance in capturing and visualizing both local and global structures of high-dimensional data.

To ensure that our data met the conditions for using UMAP, we conducted a series of analyses based on the three primary assumptions outlined by Allaoui et al. [39]. Specifically, we applied PCA for a preliminary check on the data to ensure their uniformity. We then calculated the coefficient of variation based on the mean and standard deviation of the k-NN (k-nearest neighbors) distances, obtaining a low value of approximately 0.296. Finally, we constructed a k-NN graph and found out that the data points were locally connected. The above procedures ensured the validity of our dimensionality reduction results with UMAP.

We employed the UMAP algorithm from the “umap-learn” library in Python to reduce the dimensionality of our data and used matplotlib to visualize the results, enhancing the plot with color-coded groups and annotated centroids for clarity.

## Results and discussion

### Sound symbolism across diachronic rhyme books

Different rhyme books exhibit varying levels of granularity in how characters are grouped. For example, in *GY*, characters within the same small rhyme group are listed together; several of these small rhymes then collectively form a rhyme heading group, which in turn is part of an even broader inclusive rhyme group. In contrast, *PSY* is only roughly divided into 106 rhyme categories based on the final and tone. When comparing the four rhyme books *GY*, *PSY*, *ZYYY*, and *ZHTY*, to ensure comparability, we selected the phonological unit generally referred to as *yunbu* 韻部 (rhyme category), which is primarily determined by the final and tone.

**Fig 2:**
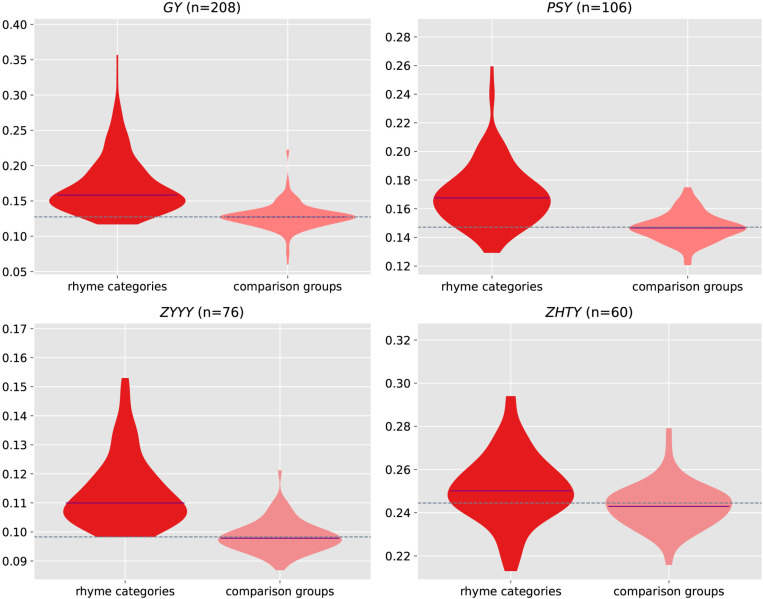
Violin plots showing the semantic similarities of rhyme categories and comparison groups across four rhyme books. Each subplot contains a left and a right violin, representing the distribution of semantic similarities of the rhyme categories and their respective comparison groups. The sample size (“n=”) indicates the number of rhyme categories in a rhyme book. The dashed line shows the average semantic similarity of the book, while the purple line within a violin marks the median of the group semantic similarities.

We created comparison groups each containing the same number of characters as the respective rhyme categories. To assess the semantic similarities of the rhyme categories and the comparison groups, we constructed the violin plots for them in Fig 2. As can be seen, in all four subplots for different rhyme books, the semantic similarities of the rhyme categories (left violins) are more vertically extended above the dashed reference line that represents the average semantic similarity of the rhyme book, resulting in taller, more peaked profiles compared to the denser and shorter distributions of comparison groups (right violins) around the reference line. This indicates generally higher semantic similarities of the rhyme categories across all four rhyme books. In the subplots, the medians of the left violins are apparently and consistently above the reference lines, while those of the right violins are very close to them, further indicating the semantic congruence of characters within the rhyme categories. This visual evidence confirms that the structured phonological groupings in these rhyme books do exhibit stronger semantic coherence.

We employed a one-sample Wilcoxon signed-rank test to examine the rhyme categories within a rhyme book. Specifically, the group-level semantic similarities, calculated as the mean of pairwise cosine similarities of characters in each rhyme category, served as the data points for the test. The book-level semantic similarity, calculated as the mean of pairwise cosine similarity across all characters covered by the book, was used as the reference value. This test assessed whether the median of the group-level semantic similarities was significantly higher than the book-level semantic similarity. We applied this test to the rhyme categories (Observed) and their respective comparison groups (Random) in all four rhyme books. We adopted a nominal significance level set at *p* = 0.05. However, to account for the eight comparisons in this study, we applied a Bonferroni correction, adjusting the significance threshold to 0.05/8 ≈ 0.0063. The statistics of these tests are presented in Table 1.

**Table 1:** Wilcoxon test statistics for the four rhyme books.

	*GY*		*PSY*		*ZYYY*		*ZHTY*	
**Book-level semantic similarity**	0.1274		0.1471		0.0983		0.2445	
**Number of groups**	208		106		76		60	
	Observed	Random	Observed	Random	Observed	Random	Observed	Random
**Mean**	0.1715	0.1278	0.1700	0.1480	0.1139	0.0987	0.2512	0.2447
**Median**	0.1583	0.1262	0.1675	0.1455	0.1100	0.0982	0.2501	0.2446
**Variance**	0.0016	0.0003	0.0005	0.0002	0.0001	0.0000	0.0003	0.0001
**W**	21562	10404	5450	2739	2925	1553	1344	901
***p*-value**	0.0000 ***	0.7033 n.s.	0.0000 ***	0.6195 n.s.	0.0000 ***	0.3206 n.s.	0.0000 ***	0.5410 n.s.

**Note**: *p *≥ 0.0063: n.s. (not significant); 0.001 ≤* p* < 0.0063: **; *p* < 0.001: ***.

The results indicate that for all four rhyme books, the rhyme categories exhibit significantly higher semantic similarity than the book-level semantic similarity, suggesting a strong phonosemantic structure encoded in the historical classification of Chinese characters in the rhyme books. In contrast, the comparison groups fail to show any significant difference from the reference values, confirming that the observed coherence in the historical categories is not an artefact of random grouping. In all cases, the W statistic for rhyme categories is notably larger than that for comparison groups, indicating greater internal semantic similarity within the rhyme categories. These statistical results reinforce the patterns observed in the violin plots, where the medians of the rhyme categories are visibly above the dotted reference lines, indicating stronger semantic coherence of these categories. The combined evidence provides compelling support for a profound correlation between sound and meaning, which is central to sound symbolism. In contrast, the comparison groups, with higher *p*-values, exhibit the expected randomness and lack of semantic coherence. This distinction proves that the sound-meaning connections are prominent only in the genuine rhyme categories, not in any randomly assembled comparison group.

As mentioned above, to overcome the limitations of solely using *Siku* embeddings for the pre-modern rhyme books, we further trained *QTS*, *QTW*, and *QTS* + *QTW* embeddings for supplementary tests on *GY* as a robustness check. To account for the eight comparisons using these four sets of embeddings, we applied a Bonferroni correction, adjusting the significance threshold to 0.05/8 ≈ 0.0063. The earlier Wilcoxon tests on the four rhyme books addressed a separate hypothesis—validating baseline semantic coherence within phonological groups—and were treated as an independent analytical stream with its own error control. This separation is to ensure that corrections for exploratory supplementary analyses do not conflate with confirmatory tests of core hypotheses, which is assumed to be the best practice for our partitioned statistical workflow. The corresponding statistics are presented in Table 2.

**Table 2:** Wilcoxon test statistics for the four embeddings on *GY.*

	*QTS*		*QTW*		*QTS *+* QTW*		*Siku*	
**Book-level semantic similarity**	0.3383		0.2895		0.3787		0.2445	
**Number of groups**	208		208		208		208	
	Observed	Random	Observed	Random	Observed	Random	Observed	Random
**Mean**	0.3564	0.3388	0.3055	0.2890	0.3962	0.3780	0.1715	0.1278
**Median**	0.3462	0.3359	0.3029	0.2861	0.3878	0.3769	0.1583	0.1262
**Variance**	0.0039	0.0032	0.0032	0.0026	0.0046	0.0028	0.0016	0.0003
**W**	14130	10474	14222	10041	13729	10537	21562	10404
***p*-value**	0.0001 ***	0.6748 n.s.	0.0001 ***	0.8293 n.s.	0.0005 ***	0.6483 n.s.	0.0000 ***	0.7033 n.s.

**Note**: *p *≥ 0.0063: n.s. (not significant); 0.001 ≤* p* < 0.0063: **; *p* < 0.001: ***.

For all four embedding sets, the rhyme categories show very low *p*-values (far below 0.0063), consistently indicating their significant semantic coherence. In contrast, the comparison groups, given their high *p*-values, show no significant semantic congruence, which aligns with the randomness in their composition. This finding reaffirms the sound-meaning connections observed in Table 1, despite the possible limitation of the character embeddings in use.

### Sound symbolism across phonological features in *GY*

**Fig 3:**
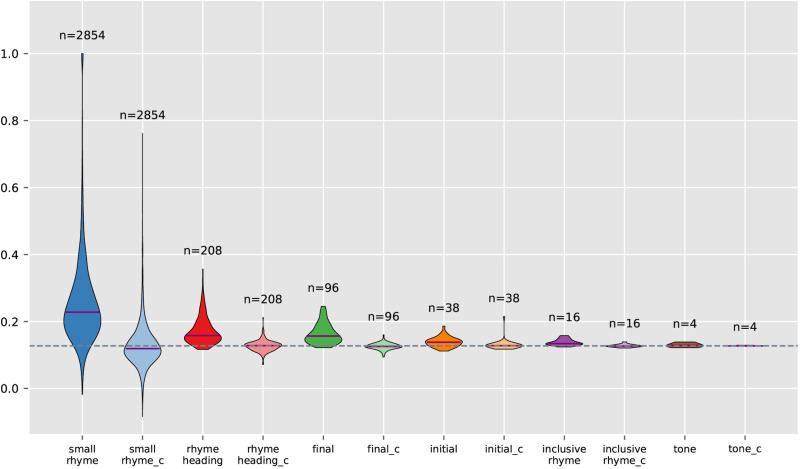
Violin plots showing the semantic similarities of phonological groups and comparison groups for *GY.* Each pair of violins represents the distribution of semantic similarities of the phonological groups and respective comparison groups, with the latter labeled with the suffix “_c” (for comparison) and displayed in a more transparent shade of the same color. The sample size (“n=”) indicates the number of phonological groups or comparison groups in a violin, where a solid purple line shows the median of the group semantic similarities. The horizontal dashed line across the whole plot marks the average semantic similarity of *GY*.

A growing body of evidence indicates that phonological elements, including vowel sounds, consonant clusters, and tones, possess inherent semantic properties that influence language perception and usage [40]. Considering this, we seek to systematically explore the symbolic capacity of various phonological units, including initials, finals, small rhymes, etc. within *GY*. For each of these phonological units, we calculated the semantic similarities of both the original phonological groups and corresponding comparison groups using the *Siku* embeddings and plotted the results in Fig 3. The number of observations incorporated in each violin may be smaller than the original count due to exclusions during the calculation process.

For the first five phonological units under study, the left violin of each pair, which represents the original phonological groups, consistently shows a median line that is distinctly higher than the dotted reference line, indicating that these groups exhibit greater semantic similarity than the whole *GY*. Meanwhile, these violin shapes are primarily skewed above the reference line, suggesting that a majority of the phonological groups have semantic similarities above the *GY* average, reinforcing the notion of sound-driven semantic coherence. In contrast, the median lines of the right violins for the comparison groups align closely with or just slightly deviate from the dotted reference line, further confirming that the observed deviation in the left violins is not an artifact of the data but a true reflection of sound-meaning associations. The distribution of the randomized comparison groups serves as a control, illustrating the random variability of group semantic similarities when a phonological relationship is absent. The stark contrast between the distributions of the original and the comparison groups substantiates the hypothesis that phonological structures in *GY* contribute substantially to semantic meanings.

For each of these five phonological units, Wilcoxon tests were conducted on the semantic similarities of the phonological groups (Observed) and those of the comparison groups (Random) against the overall average cosine similarity of *GY*, and the results are presented in Table 3. A Bonferroni correction (α = 0.05/10) was applied to account for the 10 comparisons (5 phonological units × 2 test groups: Observed and Random), ensuring strict control of the family-wise error rate.

**Table 3:** Wilcoxon test statistics for different phonological units in *GY.*

	Small rhyme		Rhyme heading		Final		Initial		Inclusive rhyme	
**Number of groups**	2854		208		96		38		16	
	Observed	Random	Observed	Random	Observed	Random	Observed	Random	Observed	Random
**Mean**	0.2587	0.1274	0.1715	0.1263	0.1638	0.1283	0.1386	0.1269	0.1378	0.1275
**Median**	0.2280	0.1174	0.1583	0.1264	0.1566	0.1275	0.1382	0.1276	0.1341	0.1279
**Variance**	0.0193	0.0039	0.0016	0.0003	0.0009	0.0002	0.0002	0.0000	0.0001	0.0000
**W**	3991816	1740021	21563	9627	4641	2303	646	362	134	76
***p*-value**	0.0000 ***	1.0000 n.s.	0.0000 ***	0.9233 n.s.	0.0000 ***	0.5364 n.s.	0.0000 ***	0.5491 n.s.	0.0000 ***	0.3529 n.s.

**Note**: *p *≥ 0.005: n.s. (not significant); 0.001 ≤* p* < 0.005: **; *p* < 0.001: ***.

The tests reveal statistically significant results for all five phonological units under study. In contrast, the comparison groups show no significant results. All these align with the visualization in Fig 3, confirming that sound-meaning association is evident across various phonological units. Notably, sound symbolism is significant not only in smaller phonological units such as initials and finals but also in broader ones like inclusive rhymes, each of which covers a wide range of characters. Its consistent presence at different phonological levels demonstrates that sound symbolism is a pervasive and integral aspect of the linguistic system.

For the tones, the Wilcoxon signed-rank test used for other phonological units is not applicable due to the limited number of groups (4 tones). Instead, we followed Otis and Sagi [19] to conduct 100 independent two-sample Welch’s t-tests for each tonal group. Each t-test compared the pairwise cosine similarities of 50 randomly sampled character pairs within a tonal group to those of 50 randomly sampled pairs from the overall population of valid characters. To ensure robustness, this process was repeated three times, and the median number of significant t-tests (uncorrected per-test *α* = 0.05) across runs was retained. To evaluate whether the observed number of significant t-tests exceeded what would be expected for random chance, we applied a binomial test. Under the null hypothesis (no systematic clustering), the number of significant t-tests should follow a binomial distribution with parameters *n* = 100 (number of t-tests) and *p* = 0.05 (baseline chance of a false positive per t-test). In order to test four tones, we applied a Bonferroni correction to account for multiple comparisons, adjusting the family-wise significance threshold to *α* = 0.0125 (0.05/4). Using exact binomial calculations based on the corrected significance level, we established the following thresholds for interpreting the results: ≥ 11 significant tests for strong evidence of clustering, 10 for marginal evidence, and ≤ 9 for no evidence. This approach balances sensitivity to tonal semantic clustering with rigorous control over family-wise error rates across all four comparisons, ensuring that the results reflect systematic patterns rather than chance fluctuations.

The statistical evaluation of the four tones revealed marked differences in their semantic coherence. We found that only the entering tone demonstrates strong evidence of systematic semantic clustering. Across three independent runs, this tone exhibited a median of 20 significant t-tests, far exceeding the precomputed threshold of 11 significant tests, suggesting that its characters share non-random semantic relationships. In contrast, the level, rising, and departing tones show no statistical support for semantic clustering, with median significant test counts of 6, 7, and 8, respectively, falling below the marginal evidence threshold of 10 significant tests. The distinctiveness of the entering tone highlights its unique role in organizing semantic information, a sound-symbolic capacity that can well account for its prominence in classical Chinese poetry. Both its distinctive phonetic profile (e.g., abrupt stops) and its semantic consistency enhance its effectiveness in the tonal patterning of *pingze* 平仄 (level and oblique tones) verse structures.

### Exemplary illustration

**Fig 4:**
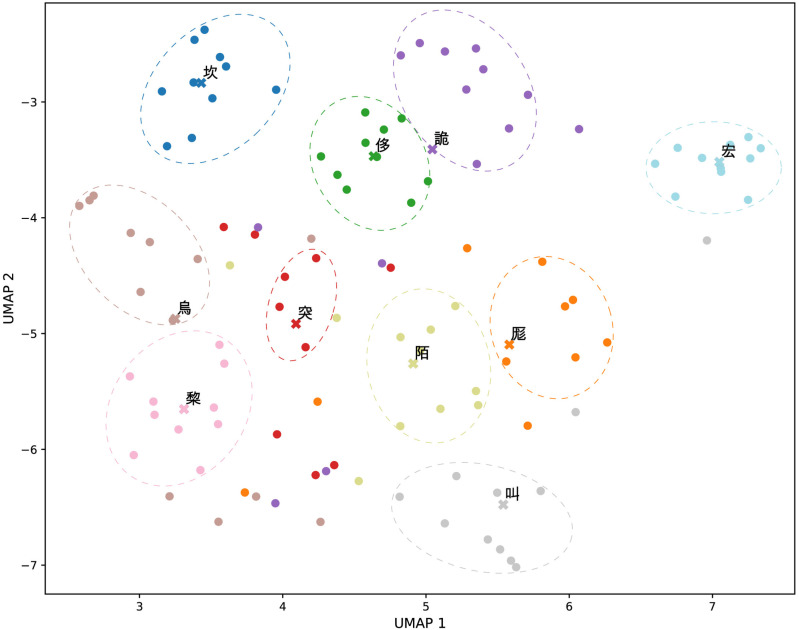
Visualization of ten top-ranked small rhyme (homophone) groups in a dimension-reduced semantic vector space. Characters from the same small rhyme group are shown in the same color. Each character is represented by a dot, and each group’s centroid by a cross, annotated with its representing character.

To achieve an intuitive demonstration of the relationship between sound and meaning, we examined homophonic characters within the same small rhyme groups in *GY*. We selected those groups containing at least 10 characters, ranked them by group semantic similarity, and chose the top 10 groups for demonstration. The character vectors then underwent dimensionality reduction with UMAP for visualization on a scatterplot as shown in Fig 4, wherein characters from the same group were color-coded identically to highlight group affiliations.

In Fig 4, homophonic characters in the same color appear close to each other and tend to form a cluster. For instance, characters in the ɣ*wœ*ŋ 宏 group, colored in light blue, form a dense cluster near the upper right corner of the plot. This clustering phenomenon indicates that homophones are semantically close to each other.

Taking a closer look at the characters of the ɣ*wœ*ŋ 宏 group, namely 宏, 紭, 嵤, 耾, 翃, 浤, 竑, 宖, 鈜, 吰, and 彋, we learned that they generally convey the meaning of “large” or “expansive”, often connected with the size of physical objects or the intensity of sounds, according to their descriptions in *GY*. For instance, 宏 literally means “large”. 紭 refers to a type of net, implying expansiveness and inclusiveness. 嵤 describes high and steep mountains, and 浤 refers to the surging and powerful movement of water. 彋 means opening and spreading out. 竑 means “measurement”, which is also size-related. 翃 refers to the flight of insects, suggesting the opening of their wings. Meanwhile, the rest characters of the group are directly related to sounds, especially loud or reverberating ones: 耾 means “whispering” according to *GY* but also often refers to thunderous sounds, 宖 refers to an echoing sound within a room, 鈜 denotes a metallic sound, and 吰 represents the sound of a bell. These characters all connect to the core theme of largeness and expansiveness, either through their literal meanings or through their associations with size and volume.

In terms of the phonetic features of the ɣ*wœ*ŋ 宏 group, the initial *ɣ* is a *hou yin* (喉音 throat sound) produced with a strong airflow, conveying a sense of depth, power, fullness, and/or vastness. The medial *w* results in a *hekou hu* 闔口呼 (rounded articulation), where the lips are rounded during pronunciation, creating a strong resonance. This resonance complements concepts like “broad” and “full”. The open front vowel *œ* is articulated with the mouth open, and when combined with the medial *w*, it enhances the rounded quality, evoking a sense of openness and expansiveness. The nasal coda *ŋ* produces an echo effect, extending and expanding the sound. Additionally, the level tone of this group is characterized by a longer duration and extended sound [41], which further enhances the effect of opening, expansion, and force. Together, these phonetic features create a sense of grandeur and openness, directly connecting to the meanings of the characters as recorded in *GY*.

The above connection between sound features and meanings of characters in the ɣ*wœ*ŋ 宏 group involves both corporeal and imitative symbolism [42]. The former is reflected in 宏, 紭, 嵤, 浤, and 彋, as their pronunciations resemble physical expansiveness, evident in the rounded *w* and the mouth opening *œ*. Concurrently, the characters 耾, 宖, 鈜, and 吰 exemplify onomatopoeia, as their pronunciations naturally mimic the loud or reverberating sounds, directly linking sound to meaning. This dual symbolism extends into more abstract terms such as 竑 (“measurement”) and 翃 (“insect flying”), illustrating how phonetic properties evolve from concrete sensory experiences to broader, more abstract concepts, effectively using physical and auditory mimicry to enrich linguistic expression and semantic depth. This shift from immediate to mediated symbolic relationships reflects the theoretical framework of mediacy versus immediacy as discussed by Jakobson and Waugh [43].

Through this exemplary analysis, we can see how the sound properties of characters in a phonological group connect to meanings, further illustrating and affirming the presence of sound symbolism. It also provides a glimpse into the underlying cognitive mechanisms driving the sound-meaning relationship of this kind, including corporeal and imitative symbolism, and their extension into more abstract concepts.

## Conclusions

This study explores sound symbolism within the Chinese language by drawing upon historical rhyme books and modern computational means. By analyzing phonological data documented over various eras, it has demonstrated that phonologically related Chinese characters exhibit semantic congruence across multiple rhyme books, underscoring the robustness of sound symbolism in the language. The granular analysis of *Guangyun* 廣韻 has further illuminated how sound symbolism permeates various levels of phonological organization. The findings confirm that sound symbolism is not an ephemeral or marginal feature of Chinese but a consistent and central element during its evolution.

The traditional rhyme books provide a valuable and reliable resource for diachronic studies of sound symbolism. By applying NLP techniques and statistical methods to this resource, this research introduces a quantitative approach to evaluating sound symbolism on a vocabulary scale, offering a more comprehensive perspective than example-based studies. These methodologies open new avenues for future research at the intersection of phonology, semantics, and language evolution. However, several of its limitations need to be acknowledged: the rhyme books primarily reflect character pronunciations in standardized language, potentially overlooking dialectal variations; the embeddings may not fully capture the nuanced meanings of characters in each rhyme book; and further tests may be required to determine whether the effect of sound symbolism is diminishing over time. Future studies are expected to expand on this groundwork by exploring additional rhyme books, employing diverse linguistic datasets, or integrating more advanced computational models to further decode the complexities of sound symbolism in Chinese and other languages. Endeavors in these directions will continue to enhance our understanding of the profound connections between sound and meaning in human language.

In terms of linguistic and philological implications, our findings inevitably engage with classical debates in Chinese character studies, such as *You Wen Shuo* 右文說 (right radical theory, i.e., using the phonetic radical on the right side of a character to interpret its meaning), by providing empirical data that inform these discussions and offer novel insights into the interplay of sound, form, and meaning in Chinese characters. By demonstrating that these correlations are systematic rather than anecdotal, we challenge the traditional dichotomy separating phonetic radicals (as purely sound-based) from semantic ones (as meaning-based), urging a re-evaluation of how sound, form, and meaning co-evolve in the writing system. Critically, this systematic, vocabulary-wide analysis—spanning the entire phonological inventory documented in rhyme books rather than in isolated subsets—reveals that sound symbolism is neither marginal nor transient. Its prevalence across phonological units (e.g., initials and finals) and historical strata underscores its durability as a key feature of language, countering the view that sound symbolism is peripheral or ephemeral. These findings not only affirm the foundational role of sound symbolism in linguistic systems but also provide empirical grounding for hypotheses about language origin and evolution, where iconicity may have served as a bridge between protolanguage’s sensory-motor mappings and modern arbitrariness. From cultural and literary perspectives, the interplay of sound and meaning invites reconsideration of poetry analysis, suggesting that classical Chinese poets may have leveraged embedded sound-meaning connections to potentially enhance conceptual coherence or tonal resonance. There is reason to invite readers to integrate phonetic patterns as a critical dimension of interpretation, uncovering stylistic subtleties that lexical or structural analysis alone has missed.

Our findings advocate for the continued exploration of sound symbolism not just as a linguistic phenomenon, but as a fundamental aspect of language that influences semantic organization and evolution. Sound symbolism offers profound insights into language as a dynamic system of meaning-making, revealing the intricate and inextricable intertwining of phonetic and semantic elements. This understanding has broad applications across various language-related fields. For example, it can enhance poetry appreciation by demonstrating how phonetic elements influence the thematic and emotional dimensions of poetry. Additionally, it can lead to intuitive language teaching methods employing phonetic cues to help students memorize word meanings by the aid of sound-meaning associations. Furthermore, it holds significant potential for improving natural language processing systems, particularly those for typical tasks like sentiment analysis and authorship attribution, by including sound-based features.
